# A case of adrenal endothelial cyst (vascular type) with positive ^123^I-MIBG uptake

**DOI:** 10.1016/j.eucr.2025.103298

**Published:** 2025-12-01

**Authors:** Riona Asai, Tomonori Sato, Yuto Yamazaki, Yuta Tezuka, Yoshikiyo Ono, Hiromichi Katayama, Yohei Satake, Takuma Sato, Yoshihide Kawasaki, Naoki Kawamorita, Hideki Katagiri, Akihiro Ito

**Affiliations:** a Department of Urology, Tohoku University Hospital, Sendai, Miyagi, Japan; b Department of Pathology, Tohoku University Hospital, Sendai, Miyagi, Japan; c Department of Diabetes, Metabolism, and Endocrinology, Tohoku University Hospital, Sendai, Miyagi, Japan

**Keywords:** Adrenal cyst, Endothelial cyst, Vascular type, MIBG, Pheochromocytoma differential diagnosis

## Abstract

^123^I-metaiodobenzylguanidine (MIBG) scintigraphy is highly accurate for diagnosing pheochromocytoma and paraganglioma (PPGL), and positive uptake in non-PPGL adrenal lesions is rare. We report a 56-year-old woman with an adrenal endothelial cyst (vascular type) showing localized ^123^I-MIBG uptake, initially suspected to be pheochromocytoma. After mild growth during observation, robot-assisted adrenalectomy was performed. Histopathology confirmed an endothelial cyst positive for CD31 and D2-40. Among 601 adrenalectomies, eight adrenal endothelial cysts (1.3 %) were identified, three showing MIBG uptake. Adrenal endothelial cysts may rarely show MIBG uptake, complicating preoperative differentiation from PPGL.

## 1 Introduction

^123^I-MIBG scintigraphy is widely used in PPGL diagnosis, with high sensitivity and specificity.^1^^,^^2^ However, physiological adrenal uptake exists, and false positives have been reported in non-PPGL adrenal lesions such as adrenocortical adenoma, schwannoma, and adrenocortical carcinoma.^3^, ^4^, ^5^ Proposed mechanisms include norepinephrine transporter (NET)-mediated uptake, vesicular monoamine transporter (VMAT)-mediated sequestration into neurosecretory granules, and passive diffusion related to increased tumor vascularity.^6^, ^7^, ^8^

According to the 2022 WHO Classification of Endocrine and Neuroendocrine Tumors (5th Edition), adrenal cysts are classified into four subtypes: pseudocyst, endothelial cyst (vascular/lymphatic type), epithelial cyst, and parasitic cyst.^9^ Endothelial cysts (vascular type) are benign, fluid-filled lesions lined by endothelial cells, usually arising from blood or lymphatic vessels. Clinically and radiologically, they may simulate cystic adrenal neoplasms, including pheochromocytoma or adrenocortical carcinoma, making accurate preoperative diagnosis challenging.^9^, ^10^, ^11^ We present the first reported adrenal endothelial cyst (vascular type) with positive ^123^I-MIBG uptake, together with review of three cases at our institution.

## 2 Case presentation

A 56-year-old woman was referred to our department after abdominal ultrasound revealed a left adrenal cyst during evaluation for hypertension detected at a health checkup. She had no remarkable past medical or family history. On presentation, her blood pressure was 144/88 mmHg and pulse rate was 68 bpm; other physical findings were unremarkable.

Laboratory findings: Plasma and urinary metanephrines and catecholamines were within normal ranges. Specifically, plasma adrenaline 20 pg/mL, plasma noradrenaline 14 pg/mL, plasma dopamine ≤20 pg/mL, urinary adrenaline 8 μg/day, urinary noradrenaline 89.1 μg/day, urinary dopamine 1100 μg/day, urinary metanephrine 0.09 mg/day, urinary normetanephrine 0.16 mg/day.

**Fig. 1:**
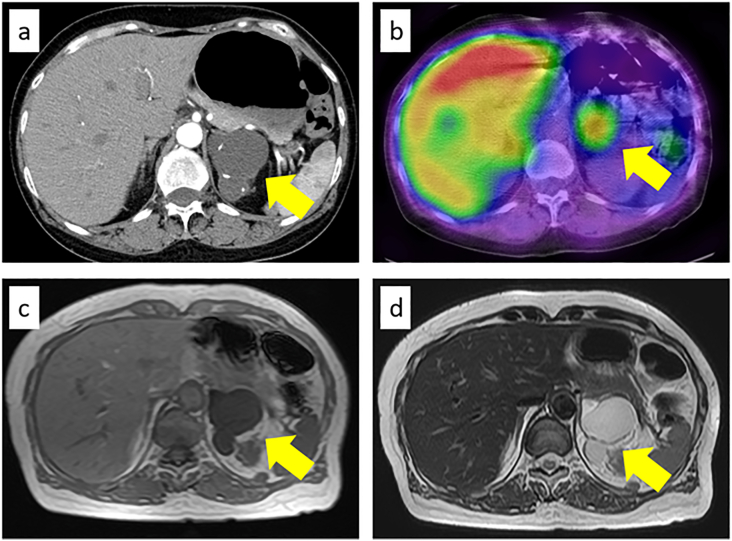
a Contrast-enhanced CT revealed a lobulated cystic lesion with calcification in the left adrenal gland. b 123I-MIBG scintigraphy showed intense uptake corresponding to the left adrenal cyst. c The lesion showed low signal intensity on T1-weighted MRI. d T2-weighted MRI demonstrated a 53-mm multilocular cystic lesion with septations and high signal intensity.

Imaging (Fig. 1): CT showed a 48-mm multilocular cystic lesion with calcification in the left adrenal gland. MRI revealed a multilocular cyst with low signal on T1-weighted and high signal on T2-weighted images. ^123^I-MIBG SPECT/CT demonstrated significant uptake at the lesion site.

Clinical course: Although pheochromocytoma was suspected, endocrine evaluations were inconclusive. The patient initially opted for observation, but gradual growth (5 mm over 2 years) led to surgical intervention. After preoperative α-blockade (doxazosin 16 mg/day), robot-assisted left adrenalectomy was performed. No intraoperative hemodynamic instability was observed. Postoperatively, no significant change in blood pressure was observed, and the pre-existing calcium-channel blocker was continued.

**Fig. 2:**
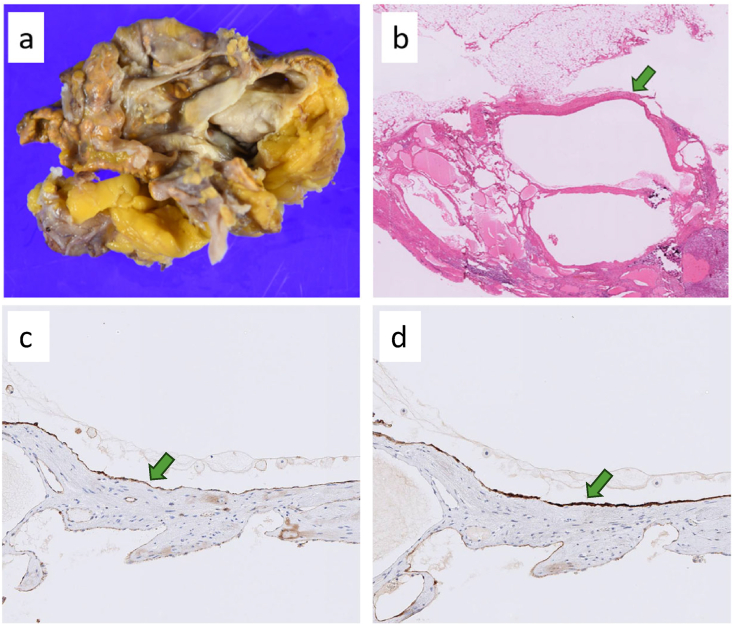
a Gross appearance of the resected specimen revealed old hemorrhagic contents within the cyst. b Histological examination showed vascular structures containing smooth muscle within the cyst wall (H&E staining, × 10). c CD31-positive endothelial cells were observed (×100). d D2-40-positive endothelial cells were also detected (×100).

Pathology: Grossly, the cyst had septations with hemorrhagic content (Fig. 2a). Histologically, the cyst wall consisted of vascular structures with smooth muscle (Fig. 2b). Endothelial cells lining the cyst wall stained positive for CD31 and D2-40 (Fig. 2c and d). The final diagnosis was adrenal endothelial cyst (vascular type).

### 2.1 Review at our institution

Among 601 adrenalectomies performed from January 2015 to January 2025, adrenal endothelial cysts (vascular type) were observed in 8 cases (1.3 %). Of these, 5 underwent ^123^I-MIBG imaging, and 3 cases (including the present case) showed positive uptake. None of the 8 cases were preoperatively diagnosed as adrenal endothelial cysts (vascular type). Surgery was performed due to suspicion of pheochromocytoma, adrenocortical carcinoma, or progressive enlargement. Clinical details of each case are summarized in Table 1.

**Table 1:** Clinical characteristics of 8 cases of adrenal endothelial cysts (vascular type).

Age	Sex	Side	Tumor size (mm)	Preoperative diagnosis	Indication for surgery	MIBG	Plasma/urinary metanephrines	Surgical approach	Immunohistochemistry
56	F	L	73 × 48	Pheochromocytoma	Suspected PPGL	Positive	Normal	Robot-assisted	CD31^+^, D2-40+
73	M	L	16 × 12	Pheochromocytoma	Suspected PPGL	Positive	Normal	Laparoscopic	N/A
77	F	L	45 × 30	Pheochromocytoma	Suspected PPGL	Positive	Abnormal	Laparoscopic	CD31^+^
30	M	L	60 × 40	Nonfunctioning adrenal tumor	Tumor size ≥40 mm	Not performed	Normal	Laparoscopic	CD31^−^, D2-40+
61	F	R	48 × 33	Nonfunctioning adrenal tumor	Tumor growth	Not performed	Normal	Laparoscopic	N/A
75	M	L	139 × 109	Nonfunctioning adrenal tumor	Tumor growth	Negative	Normal	Open	N/A
75	F	L	50 × 40	Nonfunctioning adrenal tumor	ACC not excluded	Not performed	Normal	Robot-assisted	CD31^+^
71	F	R	50 × 48	Subclinical Cushing syndrome	Tumor size ≥40 mm	Negative	Normal	Robot-assisted	CD31^+^

PPGL: pheochromocytoma and paraganglioma, ACC: adrenocortical carcinoma.

## 3 Discussion

This case highlights an adrenal endothelial cyst (vascular type) presenting with hypertension and positive ^123^I-MIBG uptake, preoperatively indistinguishable from pheochromocytoma. To our knowledge, no prior reports have described MIBG-positive adrenal endothelial cysts (vascular type). Our institutional review identified 2 additional cases, suggesting that this phenomenon should be recognized as a possible pitfall in differential diagnosis.

Endothelial cysts (vascular type) are defined histologically by the presence of endothelial lining cells within the cyst wall. Immunohistochemical positivity for CD31, CD34, and D2-40 confirms endothelial origin and distinguishes them from hematomas or mesotheliomas.^9^, ^10^, ^11^ Reported peak incidence is in the fifth to sixth decades, with a female-to-male ratio of 3:1.^9^^,^^10^ They are classified as endothelial (24–45 %), pseudocyst (39–56 %), epithelial (6–9 %), and parasitic (2–7 %).^11^ Most cases are nonfunctional and discovered incidentally. Surgical indications include suspicion of malignancy, tumor size >5 cm, hemorrhage, or symptomatic presentation. Preoperative diagnosis remains challenging, as in the present case.^10^^,^^12^

^123^I-MIBG demonstrates high sensitivity and specificity for PPGL, but physiological adrenal uptake and false positives are known. Reported non-PPGL adrenal lesions with uptake include cortical adenomas, schwannomas, and adrenocortical carcinomas. Semi-quantitative analyses (lesion-to-liver or lesion-to-mediastinum ratios) may improve diagnostic accuracy but are not definitive.^7^

Proposed mechanisms of false-positive uptake include (1) passive diffusion due to increased tumor vascularity, (2) vesicular monoamine transporter (VMAT)-mediated storage in neurosecretory granules, and (3) uptake via NET.^6^^,^^7^ In this case, passive diffusion due to vascular nature and high blood flow of the cyst was considered the most likely mechanism.

Clinical implications: Previous reports have described pheochromocytomas with normal biochemical findings, as well as cystic pheochromocytomas.^1^^,^^13^ Therefore, when surgery is considered for an MIBG-positive adrenal mass with non-typical biochemical results, adrenal endothelial cysts should also be included in the differential diagnosis. When PPGL cannot be excluded in adrenal tumors, pathological confirmation is necessary, and surgery may be unavoidable. Although biochemical findings were normal, α-blockade was administered as a precautionary measure for possible PPGL, in order to prevent perioperative blood pressure fluctuations. Preoperative α-blockade therefore remains a useful safety measure. MIBG positivity alone should not be considered diagnostic for PPGL; discrepancies with biochemical or imaging findings require careful evaluation and appropriate patient counseling.

### 3.1 Limitations

This is a single-institution, retrospective review. MIBG scintigraphy was not performed for all adrenalectomy cases, and the mechanism of false-positive uptake remains speculative.

## 4 Conclusion

Adrenal endothelial cysts (vascular type) may rarely demonstrate ^123^I-MIBG uptake, complicating differentiation from PPGL. Comprehensive diagnosis should integrate imaging, biochemical, and pathological findings.

## CRediT authorship contribution statement

**Riona Asai:** Writing – original draft, Data curation. **Tomonori Sato:** Writing – original draft, Data curation. **Yuto Yamazaki:** Writing – original draft, Investigation. **Yuta Tezuka:** Writing – original draft. **Yoshikiyo Ono:** Writing – original draft. **Hiromichi Katayama:** Data curation. **Yohei Satake:** Data curation. **Takuma Sato:** Data curation. **Yoshihide Kawasaki:** Data curation. **Naoki Kawamorita:** Data curation. **Hideki Katagiri:** Writing – review & editing, Supervision, Conceptualization. **Akihiro Ito:** Writing – review & editing, Supervision, Conceptualization.

## Funding

The publication fee of this article was supported by JSPS KAKENHI Grant Number JP24K19644.
